# Ion-Dependent ATPase Activity and Metabolic Gene Expression in TNF-α-Challenged Skeletal Muscle Cells: Mechanistic Characterisation of Carvacrol’s Bioenergetic Effects

**DOI:** 10.3390/ijms27104511

**Published:** 2026-05-18

**Authors:** Ali M. Albarrati, Rakan I. Nazer

**Affiliations:** 1 Department of Rehabilitation Sciences, College of Applied Medical Sciences, King Saud University, P.O. Box 10219, Riyadh 11433, Saudi Arabia; 2 Department of Cardiac Sciences, College of Medicine, King Saud University, Riyadh 11433, Saudi Arabia

**Keywords:** carvacrol, TNF-α, L6 myoblasts, ATPase, ion homeostasis, mitochondrial membrane potential, SIRT1, AMPK, antioxidant enzymes, inflammatory myopathy, sarcopenia, bioenergetics

## Abstract

Tumour necrosis factor-alpha (TNF-α) disrupts bioenergetic homeostasis in skeletal muscle cells through the suppression of ion-dependent ATPase activities, mitochondrial depolarisation, and impairment of antioxidant defences. Carvacrol, a phenolic monoterpenoid constituent of thyme and oregano essential oil, has been shown to exert cytoprotective effects in TNF-α-challenged L6 rat myoblasts. The mechanistic basis of these effects, specifically the relationship between membrane-associated ATPase function, mitochondrial polarisation status, and transcriptional regulation of metabolic stress-response genes, has not been formally characterised. L6 rat myoblasts were exposed to TNF-α (10 ng/mL, 1 h), then treated with carvacrol (6.25 µg/mL, 24 h) in a post-inflammatory rescue paradigm. Cell viability (MTT), membrane integrity (LDH), ion-dependent ATPase activities (Na^+^/K^+^, Ca^2+^, Mg^2+^), antioxidant enzyme activities (catalase, SOD), mitochondrial membrane potential (Muse™ MitoPotential flow cytometry), and SIRT1/AMPK mRNA expression were quantified. TNF-α significantly suppressed Na^+^/K^+^, Ca^2+^, and Mg^2+^-dependent ATPase activities (all *p* < 0.001), consistent with impaired membrane-associated bioenergetic function. Post-TNF-α carvacrol treatment partially restored all three ATPase activities (*p* < 0.05) and reduced the proportion of mitochondrially depolarised cells from 31.65 ± 4.25% to 19.0 ± 2.6% (*p* < 0.05). LDH release, catalase activity, and SOD activity were also significantly modulated. At the transcriptional level, carvacrol increased SIRT1 mRNA by 1.6-fold and AMPK mRNA by 2.0-fold relative to TNF-α-treated cells. An integrative bioenergetic model is proposed in which carvacrol’s membrane-intercalating properties restore the phospholipid environment required for ATPase conformational cycling, attenuating the Ca^2+^ overload that drives mitochondrial permeability transition, and thereby partially preserving Δψm. Transcriptional upregulation of SIRT1 and AMPKα may represent an adaptive response to residual energetic stress. The mechanistic relationships among these endpoints and the causal contribution of SIRT1 and AMPK to observed bioenergetic changes require protein-level and pathway-specific experimental validation.

## 1. Introduction

Skeletal muscle is the largest metabolically active organ in the human body, accounting for approximately 40% of total body mass and serving as the principal determinant of locomotion, glucose disposal, and systemic energy homeostasis [1,2]. Its functional integrity depends on precise maintenance of ionic gradients, mitochondrial competence, and redox balance—all of which are profoundly disrupted under conditions of chronic inflammatory stress [3,4]. Tumour necrosis factor-alpha (TNF-α), a pleiotropic pro-inflammatory cytokine whose circulating levels are elevated in age-related sarcopenia, cancer cachexia, heart failure, and post-surgical inflammatory states, is a well-characterised mediator of skeletal muscle bioenergetic dysfunction [5,6]. At the cellular level, TNF-α activates NF-κB via reactive oxygen species (ROS)-dependent signalling, suppresses mitochondrial oxidative phosphorylation efficiency, promotes inner membrane depolarisation, and impairs the ion-transporting ATPases that maintain electrochemical gradients essential for membrane excitability and contraction [7,8].

Among the cellular systems most sensitive to TNF-α-mediated bioenergetic insult, ion-dependent ATPases occupy a uniquely informative position. Na^+^/K^+^-ATPase sustains the trans-sarcolemmal sodium and potassium gradients fundamental to action potential generation and excitation–contraction coupling; its suppression under inflammatory conditions directly impairs force production and fatigability [9]. Ca^2+^-ATPase regulates cytosolic and sarcoplasmic reticulum calcium dynamics essential for contraction–relaxation cycling; its impairment by ROS-mediated cysteine oxidation compromises calcium homeostasis and promotes apoptotic signalling [10]. Mg^2+^-ATPase, representing composite cellular ATP-hydrolysing capacity in the presence of Mg^2+^, serves as a general bioenergetic indicator reflecting the integrated energy metabolism of the cell [11]. Characterising the response of all three ion-dependent ATPase systems to inflammatory challenge and to pharmacological intervention provides a multi-dimensional window into membrane bioenergetic function that single-endpoint approaches cannot capture.

The energy-sensing serine/threonine kinase AMPK and the NAD^+^-dependent protein deacetylase SIRT1 function as an integrated metabolic stress-response axis that is mechanistically connected to both mitochondrial function and ATPase-dependent ion homeostasis [12,13]. AMPK, activated by elevated AMP:ATP ratios resulting from bioenergetic stress, phosphorylates multiple downstream targets including PGC-1α, mitochondrial biogenesis, ULK1, mitophagy initiation, and ACC, fatty acid oxidation [12]. SIRT1, whose activity depends on NAD^+^ availability—itself a function of the cell’s redox and energetic state—deacetylates PGC-1α and FOXO3a, promoting antioxidant gene expression and mitochondrial quality control [13]. The reciprocal relationship between AMPK and SIRT1 creates a self-reinforcing adaptive response to energetic stress that, if intact, could theoretically support recovery of ATPase function and mitochondrial polarisation following inflammatory injury. Whether transcriptional upregulation of these genes by carvacrol reflects functional activation of this axis, or represents a transcriptional response that is not translated into downstream enzymatic activity, is a central mechanistic question that requires protein-level investigation.

Carvacrol, a phenolic monoterpenoid constituting up to 80% of thyme and oregano essential oil, possesses well-documented antioxidant, anti-inflammatory, and membrane-active properties [14,15]. Its lipophilic monoterpenoid backbone enables direct integration into phospholipid bilayers, modulating membrane fluidity and the conformational dynamics of membrane-associated enzymes including ATPases [14]. Carvacrol also activates PPARα and PPARγ, suppresses NF-κB-driven inflammatory signalling, and has been proposed to modulate the SIRT1/AMPK axis in hepatic and neural cellular models [15,16]. A preliminary report from our previous study demonstrated that carvacrol at 6.25 µg/mL exerts cytoprotective effects in TNF-α-challenged L6 rat myoblasts, attenuating cytotoxicity, LDH release, and mitochondrial depolarisation, and upregulating SIRT1 and AMPK mRNA [17]. However, the mechanistic basis of the observed effects, specifically how carvacrol influences ion-dependent ATPase function in relation to mitochondrial polarisation, how these endpoints relate to each other mechanistically, and what molecular events underlie the transcriptional changes in SIRT1 and AMPK was not addressed in that report.

Therefore, the aim of the present study was to investigate whether carvacrol modulates cellular bioenergetic and stress-response pathways in TNF-α-challenged L6 rat myoblasts. We hypothesised that carvacrol would partially attenuate TNF-α–induced bioenergetic dysfunction by modulating ion-dependent ATPase activity, mitochondrial polarisation, antioxidant enzyme function, and transcriptional regulation of metabolic stress-response genes.

## 2. Results

### 2.1. Carvacrol Viability and Concentration Selection

**Figure 1:**
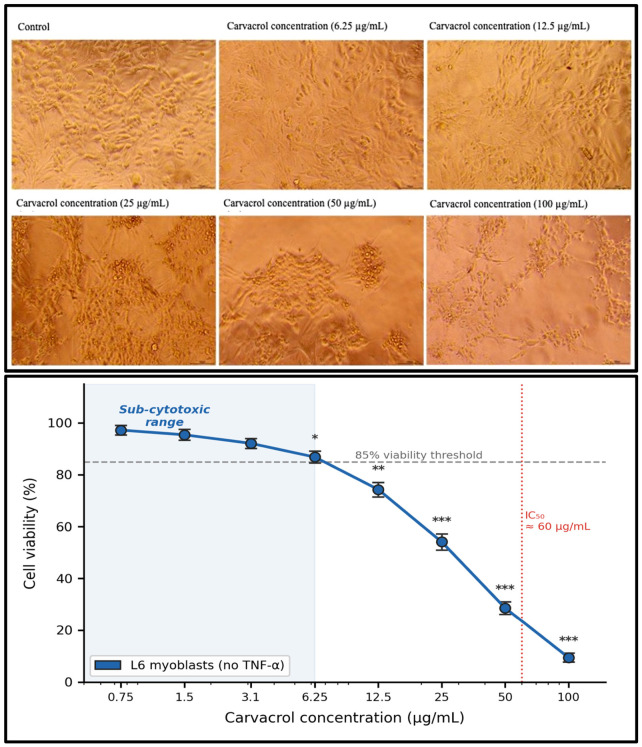
Concentration-dependent cytotoxicity of carvacrol in L6 rat myoblasts. Cells were treated with increasing concentrations of carvacrol (0.75–100 µg/mL) for 24 h in the absence of TNF-α, and viability was assessed using the MTT assay. Note the mild but consistent reduction in viability at 6.25 µg/mL relative to control, and the marked concentration-dependent decline above 12.5 µg/mL approaching the IC_50_ (∼60 µg/mL). Data are presented as mean ± SEM (n = 3). *** *p* < 0.001 vs. control; ** *p* < 0.01 vs. control; * *p* < 0.05 vs. control (one-way ANOVA, Tukey’s HSD).

**Figure 2:**
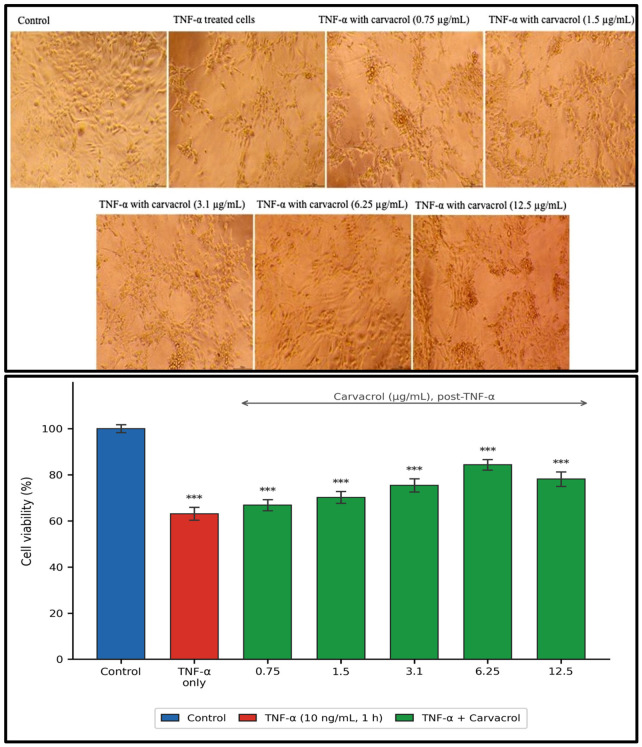
Cytoprotective effect of post-TNF-α carvacrol treatment on L6 myoblast viability. Cells were pre-exposed to TNF-α (10 ng/mL, 1 h) to induce inflammatory stress, followed by treatment with carvacrol at the indicated concentrations for 24 h. Cell viability was assessed using the MTT assay. Data are presented as mean ± SEM (n = 3). *** *p* < 0.001 vs. untreated control (one-way ANOVA, Tukey’s HSD).

Carvacrol produced a concentration-dependent reduction in L6 viability (IC_50_ = 60.87 µg/mL [1]; Figure 1). The working concentration of 6.25 µg/mL maintained viability at 87.3 ± 2.1% of vehicle control, a mild intrinsic sub-cytotoxic effect (~12.7% reduction) that is explicitly acknowledged as a design constraint throughout. Sequential TNF-α pre-treatment (10 ng/mL, 1 h) significantly reduced viability (*p* < 0.001; partial η^2^ = 0.94); carvacrol at 6.25 µg/mL produced the greatest attenuation of TNF-α-associated viability loss across the tested concentration range (Figure 2).

### 2.2. Ion-Dependent ATPase Activities: Quantitative Profile

**Figure 3:**
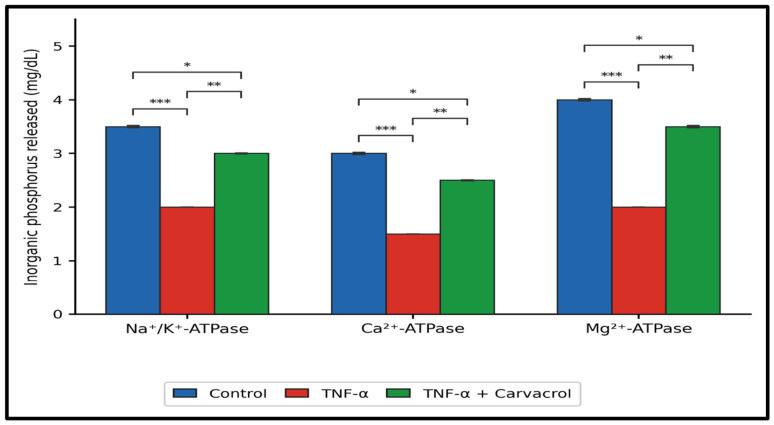
Ion-dependent ATPase activities following TNF-α exposure and carvacrol treatment. L6 myoblasts were exposed to TNF-α (10 ng/mL, 1 h) followed by carvacrol (6.25 µg/mL, 24 h). Activities of Na^+^/K^+^-dependent, Ca^2+^-dependent, and Mg^2+^-dependent ATPases were assessed as ion-dependent ATP hydrolysis capacity by inorganic phosphate release. Data are mean ± SEM (n = 3). *** *p* < 0.001; ** *p* < 0.01; * *p* < 0.05 (one-way ANOVA, Tukey’s HSD).

TNF-α exposure significantly reduced all three ion-dependent ATPase activities relative to untreated controls (*p* < 0.001; Figure 3). Na^+^/K^+^-ATPase activity decreased from 3.5 ± 0.02 to 2.0 ± 0.001 mg/dL; Ca^2+^-ATPase from 3.0 ± 0.02 to 1.5 ± 0.001 mg/dL; and Mg^2+^-ATPase from 4.0 ± 0.02 to 2.0 ± 0.001 mg/dL. These reductions are consistent with ROS-mediated oxidative inactivation of ATPase active-site thiols and altered membrane lipid composition reducing enzyme conformational mobility under inflammatory conditions.

Post-TNF-α carvacrol treatment (6.25 µg/mL) significantly restored all three activities relative to TNF-α alone (Figure 3): Na^+^/K^+^ to 3.0 ± 0.01 mg/dL (*p* < 0.05); Ca^2+^ to 2.5 ± 0.01 mg/dL (*p* < 0.05); and Mg^2+^ to 3.5 ± 0.02 mg/dL (*p* < 0.01). Importantly, all three activities remained significantly below control values (*p* < 0.05 for Na^+^/K^+^ and Mg^2+^; *p* < 0.01 for Ca^2+^), indicating partial rather than complete functional recovery. The pattern of ATPase suppression and partial recovery was consistent across all three ionic conditions, suggesting that carvacrol’s effects on membrane bioenergetics are not ion-specific but reflect a more general stabilisation of the membrane environment in which these enzymes operate.

### 2.3. LDH Release and Membrane Integrity

**Figure 4:**
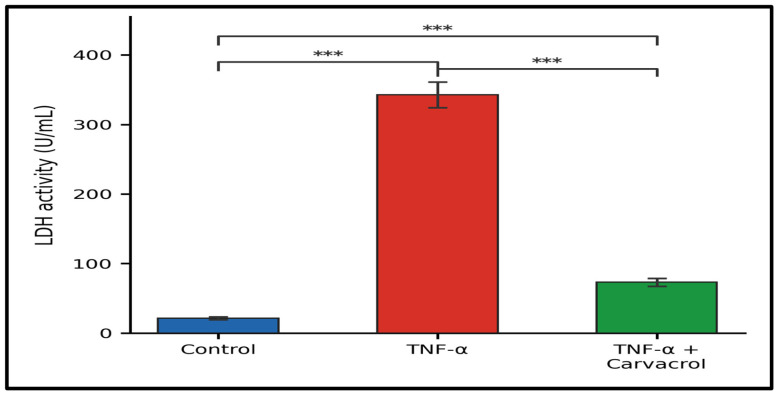
LDH release following TNF-α exposure and carvacrol treatment. L6 myoblasts were exposed to TNF-α (10 ng/mL, 1 h) followed by carvacrol (6.25 µg/mL, 24 h). LDH activity in culture supernatants was quantified as an index of membrane damage. Data are mean ± SEM (n = 3). *** *p* < 0.001 (one-way ANOVA, Tukey’s HSD).

TNF-α markedly increased LDH activity in culture supernatants relative to controls (*p* < 0.001; Figure 4), confirming plasma membrane disruption. Post-TNF-α carvacrol reduced LDH release significantly (*p* < 0.001), though residual LDH activity remained above control levels, consistent with partial membrane protection. These findings are mechanistically relevant to the ATPase data: plasma membrane disruption detected by LDH release would independently compromise Na^+^/K^+^-ATPase function by dissipating the ionic gradients against which this enzyme operates, making LDH and ATPase recovery closely coupled indices of membrane integrity restoration.

### 2.4. Antioxidant Enzyme Activities

**Figure 5:**
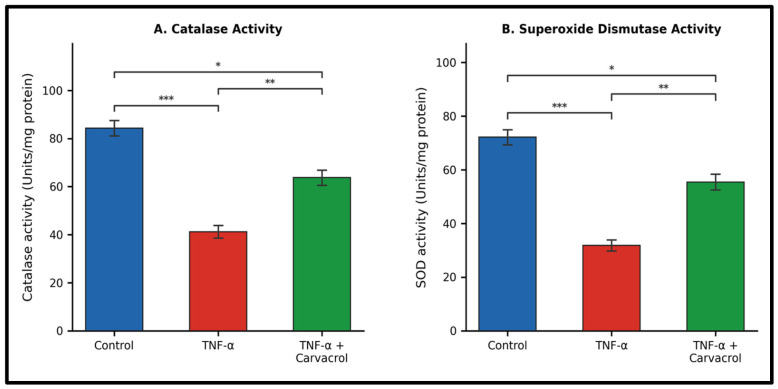
Catalase and superoxide dismutase (SOD) activities following TNF-α exposure and carvacrol treatment. L6 myoblasts were exposed to TNF-α (10 ng/mL, 1 h) followed by carvacrol (6.25 µg/mL, 24 h). Enzyme activities were normalised to total protein content (Bradford assay). Data are mean ± SEM (n = 3). *** *p* < 0.001; ** *p* < 0.01; * *p* < 0.05 (one-way ANOVA, Tukey’s HSD).

Catalase and SOD activities were significantly reduced following TNF-α exposure relative to untreated controls (Figure 5). Specifically, TNF-α treatment was associated with reduced catalase and SOD activities (*p* < 0.001 for both), consistent with antioxidant system impairment under inflammatory stress. Post-TNF-α carvacrol treatment was associated with significant increases in both catalase and SOD activities relative to TNF-α alone (*p* < 0.005 for both). Despite this partial recovery, enzyme activities did not fully return to control levels.

### 2.5. Mitochondrial Membrane Potential

**Figure 6:**
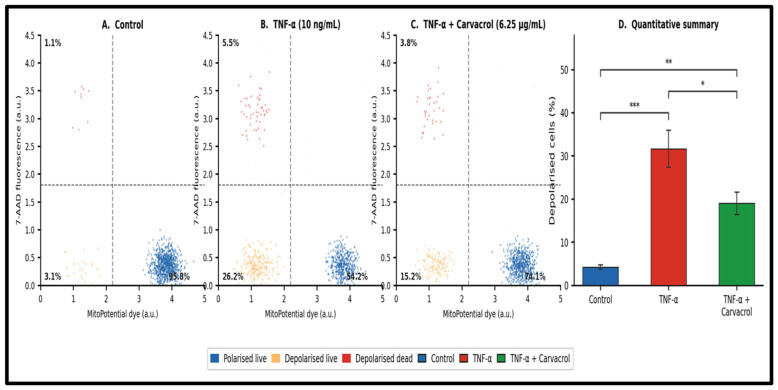
Mitochondrial membrane potential analysis following TNF-α exposure and carvacrol treatment. (**A**–**C**) Representative two-parameter Muse™ MitoPotential dot plots (MitoPotential dye fluorescence versus 7-AAD) for each experimental condition (Control, TNF-α, TNF-α + Carvacrol). Quadrant gating discriminates polarised live (upper left), depolarised live (lower left), and depolarised dead (lower right) populations. (**D**) Quantitative summary of the percentage of depolarised cells (mean ± SEM, n = 3) across all three conditions. *** *p* < 0.001 Control vs. TNF-α; ** *p* < 0.01 Control vs. TNF-α + Carvacrol; * *p* < 0.05 TNF-α vs. TNF-α + Carvacrol (one-way ANOVA, Tukey’s HSD).

Muse™ MitoPotential flow cytometry demonstrated that TNF-α exposure significantly increased the proportion of mitochondrially depolarised cells from a baseline range of approximately 4–5% (control) to 31.65 ± 4.25% (*p* < 0.001; Figure 6), consistent with TNF-α-induced ROS production promoting inner mitochondrial membrane potential (Δψm) dissipation. Post-TNF-α carvacrol treatment reduced the depolarised fraction to 19.0 ± 2.6% (*p* < 0.05 vs. TNF-α; Figure 6D), indicating partial preservation of Δψm. The carvacrol-treated group remained significantly above control levels (*p* < 0.01), confirming incomplete mitochondrial protection at this concentration. Representative dot plots for each condition are provided in Figure 6A–C to permit an independent assessment of gating quality.

### 2.6. SIRT1 and AMPKα mRNA Expression

**Figure 7:**
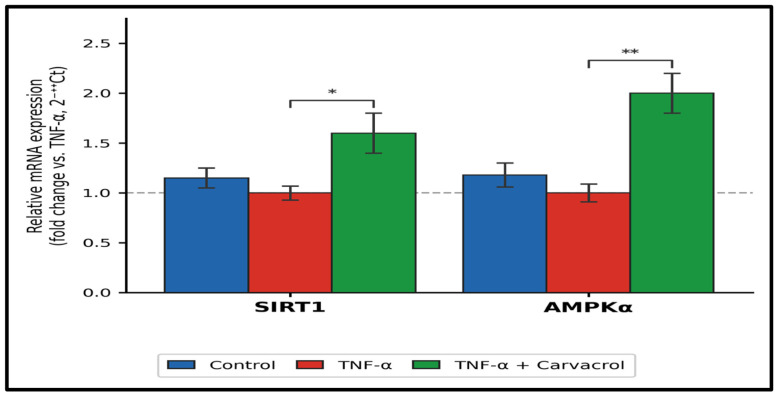
Relative mRNA expression of SIRT1 and AMPK following TNF-α exposure and carvacrol treatment. L6 myoblasts were exposed to TNF-α (10 ng/mL, 1 h) followed by carvacrol (6.25 µg/mL, 24 h). mRNA expression was quantified by quantitative RT-PCR using the 2^−ΔΔCt^ method and normalised to GAPDH. Data are expressed as fold change relative to the TNF-α-treated group and presented as mean ± SEM (n = 3). Significance level: ** *p* < 0.01; * *p* < 0.05 (one-way ANOVA, Tukey’s HSD).

Quantitative RT-PCR analysis demonstrated that TNF-α exposure modestly downregulated SIRT1 and AMPK mRNA levels relative to untreated controls (Figure 7). Post-TNF-α carvacrol treatment was associated with significant increases in SIRT1 mRNA (1.62 ± 0.18-fold; *p* < 0.05) and AMPK mRNA (2.04 ± 0.23-fold; *p* < 0.01) relative to TNF-α-treated cells. These findings represent gene transcription-level observations quantified by the 2^−ΔΔCt^ method relative to GAPDH. They do not establish changes in SIRT1 or AMPK protein abundance, post-translational modification status, or functional enzymatic activity.

## 3. Discussion

The present study provides a quantitative mechanistic characterisation of carvacrol’s effects on bioenergetic function in TNF-α–challenged skeletal muscle cells. The results demonstrate that post-TNF-α treatment with carvacrol was associated with partial attenuation of cytotoxicity and LDH release, partial recovery of ion-dependent ATPase activities, reduced mitochondrial depolarisation, altered antioxidant enzyme activities, and increased mRNA expression of the metabolic stress-response genes SIRT1 and AMPK.

The suppression of Na^+^/K^+^, Ca^2+^, and Mg^2+^-dependent ATPase activities by TNF-α is mechanistically explicable through two complementary pathways. First, TNF-α-driven mitochondrial ROS production oxidatively modifies the reactive cysteine and methionine residues within the nucleotide-binding and phosphorylation domains of P-type ATPases, directly impairing their hydrolytic capacity [10,11]. Second, TNF-α alters membrane phospholipid composition by promoting ceramide generation, increasing bilayer saturation, and reducing the fluidity required for the large conformational changes that ATPases must undergo during their catalytic cycle [12,18]. Carvacrol’s partial reversal of these suppressive effects is consistent with both its documented capacity to intercalate into phospholipid bilayers and restore membrane fluidity [15] and its anti-inflammatory properties that may attenuate ROS generation by partially suppressing NF-κB signalling [16,19]. The parallel partial recovery across all three ionic conditions, rather than ion-specific rescue, supports the interpretation that carvacrol acts primarily on the shared membrane environment rather than on individual enzyme species.

A mechanistically important observation is the parallel partial recovery of LDH release and ATPase activities. LDH leakage reflects plasma membrane disruption—the same membranous compartment in which Na^+^/K^+^-ATPase is embedded and operates. Membrane damage that allows LDH release would independently dissipate the transmembrane ionic gradients against which Na^+^/K^+^-ATPase works, thereby reducing apparent enzyme activity even if the enzyme itself were unaffected. The parallel proportional recovery of both parameters under carvacrol treatment therefore implies that a shared membrane-structural restoration mechanism underlies both effects, specifically, that carvacrol’s membrane-intercalating properties physically seal or stabilise the sarcolemma, simultaneously preventing LDH efflux and restoring the electrochemical gradients within which Na^+^/K^+^-ATPase can function. This membrane-ATPase structural linkage has not previously been articulated in the carvacrol literature and representing a novel contribution of the present study.

The partial preservation of mitochondrial membrane potential by carvacrol is consistent with the suppression of TNF-α-driven mitochondrial permeability transition pore (mPTP) opening, which is initiated by elevated matrix Ca^2+^ and ROS [20,21]. By partially restoring Ca^2+^-ATPase activity, carvacrol may indirectly attenuate the cytosolic and mitochondrial Ca^2+^ overload that promotes mPTP opening, creating a mechanistic link between Ca^2+^-ATPase recovery and mitochondrial polarisation that is independently plausible but requires direct experimental testing using Ca^2+^ chelation and mPTP pharmacological probes. The incomplete nature of Δψm recovery at 6.25 µg/mL is consistent with the incomplete suppression of upstream ROS generation and the narrow effective concentration window of carvacrol observed in the cytotoxicity profile.

TNF-α promotes mitochondrial superoxide production primarily at Complex I, NADH:ubiquinone oxidoreductase, through reverse electron transport and at Complex III, ubiquinol:cytochrome c oxidoreductase, through the ubisemiquinone radical mechanism [4,5]. Superoxide dismutation to H_2_O_2_ creates an oxidative burden that inactivates antioxidant enzymes, catalase is susceptible to inactivation by H_2_O_2_ at concentrations above 1 mM; SOD is susceptible to peroxynitrite generated by NO/superoxide interaction, and simultaneously oxidises ATPase reactive cysteines [9,20]. The partial recovery of catalase and SOD activities following carvacrol treatment is mechanistically consistent with two non-mutually exclusive hypotheses. Firstly, reduced ROS generation secondary to partial NF-κB suppression by carvacrol, which would reduce the oxidative burden driving antioxidant enzyme inactivation. Secondly, transcriptional induction of antioxidant gene expression mediated by carvacrol-activated PPARα and potentially Nrf2, both of which drive catalase and SOD gene transcription [14,22]. Critically, because a direct ROS measurement was not performed, we cannot determine which mechanism predominates or whether carvacrol meaningfully reduces cellular oxidative burden at 6.25 µg/mL. This is an important interpretive gap: the observed antioxidant enzyme changes could theoretically represent compensatory upregulation in a cell still under sustained oxidative stress, rather than evidence of ROS reduction. Resolving this requires concurrent DCFH-DA-based total ROS quantification and MitoSOX-based mitochondrial superoxide measurement in future studies.

The transcriptional upregulation of SIRT1 and AMPKα mRNA by carvacrol represents a potentially significant but strictly mRNA-level observation [23]. AMPK activation is fundamentally governed post-translationally: the kinase is activated by phosphorylation at Thr172 of the catalytic α-subunit by the upstream kinases LKB1 (constitutive) and CaMKKβ (Ca^2+^-dependent) in response to elevated AMP:ATP ratios [11]. Increased AMPK mRNA does not establish Thr172 phosphorylation, nor does it confirm phosphorylation of downstream substrates including ACC (Ser79) and ULK1 (Ser555), which are required to demonstrate functionally active AMPK signalling [11,12]. Similarly, SIRT1 deacetylase activity is obligatorily dependent on intracellular NAD^+^ availability, which was not measured in this study, and SIRT1 protein abundance is regulated by proteasomal degradation independently of mRNA levels. Transcriptional upregulation of SIRT1 mRNA therefore does not establish deacetylation of canonical SIRT1 substrates including PGC-1α, FOXO3a, and NF-κB p65 [11]. All SIRT1 and AMPKα findings in this study are therefore explicitly limited to the transcriptional level.

The novel mechanistic contribution of the present study lies in the proposed integration of these endpoints into a coherent bioenergetic framework. Carvacrol’s membrane-intercalating properties plausibly explain its direct effects on ATPase activity and membrane integrity simultaneously. Recovery of ATPase function reduces the energetic cost of maintaining ionic homeostasis, potentially reducing the ATP:AMP ratio depression that drives AMPK activation. Conversely, transcriptional AMPK upregulation—if translated into kinase activation—would promote PGC-1α-dependent mitochondrial biogenesis and antioxidant gene expression, establishing a feed-forward loop that could sustain the partial bioenergetic recovery observed. Whether this integration represents a causal hierarchy (membrane effects → AMPK → mitochondria → ATPase) or a set of parallel but mechanistically independent responses to carvacrol’s pharmacological properties requires pathway-specific dissection using compound C (AMPK inhibitor), sirtinol (SIRT1 inhibitor), and GW6471 (PPARα antagonist) in conjunction with protein level endpoints.

The present findings are consistent with and mechanistically extend our previous study [17], which demonstrated the cytoprotective potential of carvacrol across multiple functional endpoints for a pharmacological audience. The present analysis provides the mechanistic framework, specifically the ATPase bioenergetic linkage, the membrane-ATPase-mitochondria mechanistic integration, and the protein-validation requirement for SIRT1/AMPK conclusions that was not addressed in that preliminary report. Together, the two studies provide complementary perspectives on the same biological phenomenon: one establishing that the effect exists and has therapeutic relevance, the other characterising its mechanistic basis and identifying the specific experimental steps required to establish causal relationships.

Several limitations constrain the mechanistic conclusions that can be drawn from the present study. First, the absence of a carvacrol-only control arm, carvacrol without TNF-α, across all endpoints prevents the attribution of the observed changes to cytoprotection against TNF-α versus direct pharmacological modulation of basal L6 myoblast physiology. This is the most consequential design constraint, and a full 2 × 2 factorial design (±TNF-α × ±carvacrol) is the primary experimental modification of the planned follow-up study. Second, the model uses undifferentiated L6 myoblasts, which differ substantially from post-mitotic skeletal muscle fibres in ATPase isoform composition, mitochondrial density, and inflammatory signalling architecture; validation in differentiated L6 myotubes and primary human satellite cell-derived myotubes is required. Third, a direct ROS measurement was not performed. Fourth, SIRT1 and AMPK conclusions are limited to the mRNA level. Fifth, no positive pharmacological controls (AICAR for AMPK; resveratrol for SIRT1; N-acetylcysteine for ROS) were included to contextualise the magnitude of observed responses.

## 4. Materials and Methods

### 4.1. Cell Culture

L6 rat skeletal myoblasts (ATCC CRL-1458; RRID:CVCL_0385) were cultured in RPMI-1640 medium (Sigma-Aldrich, St. Louis, MO, USA) supplemented with 10% heat-inactivated foetal bovine serum (FBS) and 1% penicillin–streptomycin. Cells were maintained at 37 °C in a humidified 5% CO_2_ atmosphere and passaged between passages 8 and 15 to minimise phenotypic drift. All experiments were initiated at 70–80% confluence.

### 4.2. Carvacrol Cytotoxicity Profile (MTT Assay)

L6 cells (10,000 cells/well) were seeded in 96-well plates and incubated overnight to permit attachment. Cells were then exposed to increasing concentrations of carvacrol (0.75, 1.5, 3.1, 6.25, 12.5, 25, 50, and 100 µg/mL) for 24 h. MTT solution (10 µL, 5 mg/mL in phosphate-buffered saline) was added per well and incubated for 4 h at 37 °C in the dark. After removal of media, DMSO (100 µL) was added to dissolve formazan crystals, and absorbance was measured at 570 nm using a BioTek Synergy HT plate reader. Cell viability was calculated as:

Cell viability (%) = (Mean absorbance treated/Mean absorbance control) × 100.

Concentration selection for downstream mechanistic experiments was based on identifying a sub-IC_50_ dose that preserved sufficient cellular metabolic activity for valid enzymatic and molecular analyses, while demonstrating measurable attenuation of TNF-α–induced cytotoxicity. A concentration of 6.25 µg/mL was selected on the basis that: (i) it is approximately 10-fold below the estimated IC_50_ (∼60 µg/mL); (ii) it maintained viability above 85% relative to untreated controls in the absence of TNF-α; (ii) it demonstrated the greatest relative attenuation of TNF-α-associated viability loss among tested concentrations; and (iii) concentrations ≥ 12.5 µg/mL produced concentration-dependent baseline cytotoxicity that would confound interpretation of cytoprotective effects [17].

### 4.3. Post-TNF-α Cytoprotective Assay

Cells were pre-exposed to TNF-α (10 ng/mL, 1 h) to establish inflammatory stress, then treated with ascending concentrations of carvacrol (0.75, 1.5, 3.1, 6.25, and 12.5 µg/mL, 24 h). This sequential post-inflammatory protocol models therapeutic intervention after stress onset, consistent with the clinical scenarios in which pharmacological agents are administered to patients with established inflammatory conditions [5,6,17]. Experimental groups: (i) vehicle control; (ii) TNF-α alone; (iii) TNF-α + carvacrol at each concentration. Viability was quantified by MTT as above. Cell morphology was documented by phase-contrast microscopy at each condition.

### 4.4. Lactate Dehydrogenase (LDH) Assay

Cells were pre-treated with TNF-α (10 ng/mL, 1 h) and subsequently exposed to carvacrol (6.25 µg/mL, 24 h). Culture supernatants were collected and analysed using a commercial LDH assay kit (Coral Clinical Systems, Gaa, India; Cat. No. 110410025). Briefly, 50 µL of supernatant was mixed with 1 mL of working reagent, and absorbance at 340 nm was measured after 1 min. LDH activity was calculated as:

LDH activity (U/mL) = ΔOD/min × 3333

The conversion constant (3333) is the assay-specific factor derived from the molar extinction coefficient of NADH (6220 M^−1^cm^−1^), the optical path length, and the reaction and sample volumes according to the kit protocol. Results are expressed as LDH activity (U/mL) and interpreted as an index of membrane damage.

### 4.5. Cell Lysis and Protein Quantification

Following treatment, cells were detached by trypsinisation, collected by centrifugation at 5000× *g* for 15 min at 4 °C, and lysed in ice-cold lysis buffer (0.1 M Tris-HCl, pH 7.4; 0.2 M EDTA; 2 M NaCl; 0.5% Triton X-100) for 20 min at 4 °C with periodic vortexing. The buffer components serve the following functions: Tris-HCl (0.1 M, pH 7.4) maintains physiological pH during lysis; EDTA (0.2 M) chelates divalent cations to inhibit metalloprotease activity and prevent non-enzymatic ATP hydrolysis; NaCl (2 M) maintains ionic strength to stabilise protein conformation; and Triton X-100 (0.5%) disrupts phospholipid bilayers to release cytosolic and membrane-associated enzyme fractions. Lysates were clarified by centrifugation at 10,000× *g* for 10 min at 4 °C to remove cellular debris. Total protein concentration was determined in the supernatant using the Bradford colorimetric assay, and all enzyme activities were normalised to total protein content and expressed as units per milligram of protein.

### 4.6. Antioxidant Enzyme Assays

#### 4.6.1. Catalase Activity

Cell lysate (0.5 mL) was combined with 1.2 mL of 0.01 M sodium phosphate buffer (pH 7.0) in a UV-transparent cuvette. The reaction was initiated by adding 1.0 mL of freshly prepared 0.2 mM hydrogen peroxide (H_2_O_2_) solution. Absorbance at 240 nm was recorded every 30 s for 3 min at 25 °C, exploiting the characteristic UV absorbance of H_2_O_2_ (ε_240_ = 43.6 M^−1^cm^−1^). Catalase activity was calculated as:

Catalase (Units/mL) = [(ΔA_blank − ΔA_sample) × d]/(V × 0.043).

where ΔA represents the change in absorbance per minute, d is the dilution factor, V is the sample volume in mL, and 0.043 is the molar extinction coefficient-derived constant for H_2_O_2_ at 240 nm (ε_240_ = 43.6 M^−1^cm^−1^ × 10^−3^ to convert from M^−1^ to mM^−1^ units). One unit of catalase activity is defined as the amount of enzyme required to decompose 1 µmol H_2_O_2_ per minute at 25 °C and pH 7.0.

#### 4.6.2. Superoxide Dismutase (SOD) Activity

SOD activity was determined by the nitro blue tetrazolium (NBT) photochemical reduction method [24]. Briefly, 50 µL of cell lysate was added to a reaction mixture containing 50 mM sodium phosphate buffer (pH 7.8), 45 µM methionine, 5.3 mM riboflavin, 84 µM potassium ferricyanide, and 0.3 mM NBT in a final volume of 3 mL. Samples were exposed to a fluorescent light source (15 W, 30 cm distance) for exactly 10 min at 25 °C to drive riboflavin-mediated photoreduction of NBT, which generates a formazan product detectable at 600 nm. Dark-incubated blanks (no light exposure) confirmed background NBT reduction. Absorbance at 600 nm was measured immediately at the end of the irradiation period. SOD activity was quantified using:

% inhibition = [(Control − Test)/Control] × 100.

SOD units (U/mg protein) = % inhibition/50.

One unit of SOD activity is defined as the amount of enzyme causing 50% inhibition of NBT photoreduction under the described assay conditions. All measurements were conducted in triplicate and normalised to total protein concentration determined by the Bradford assay.

### 4.7. Ion-Dependent ATPase Activity Assays

#### 4.7.1. Interpretive Framework

Ion-dependent ATPase activities were assessed by measuring inorganic phosphate (Pi) release following ATP hydrolysis in the presence of defined ionic conditions. In the present study, selective pharmacological inhibitors, including ouabain to isolate the ouabain-sensitive fraction of Na^+^/K^+^-ATPase or N-ethylmaleimide (myosin ATPase alkylating agent) were not employed. Consequently, the measured activities represent total ion-dependent ATP hydrolysis under the respective ionic conditions rather than absolute isoform-specific enzyme activities. Multiple ATPases may contribute to Pi release under each assay condition, and cross-contamination between enzymatic contributions cannot be formally excluded. In particular, Mg^2+^ serves as a universal cofactor for nucleoside triphosphate hydrolysis and is required by virtually all ATPase enzymes; the Mg^2+^-dependent activity measurement therefore represents a composite indicator of cellular ATP-hydrolysing capacity rather than a specific enzymatic entity.

#### 4.7.2. Inorganic Phosphate Quantification

Pi release was quantified using the Erba Phosphorus Reagent Kit (Erba Diagnostics Mannheim GmbH, Mannheim, Germany, #120226), which is based on the phosphomolybdate colorimetric reaction. Briefly, 1000 µL of working reagent was added to 20 µL of sample, reagent blank (distilled water), or the supplied phosphorus standard. After incubation for 5 min at 37 °C, absorbance was recorded at 340 nm. Phosphorus concentration was calculated as:

Phosphorus (mg/dL) = (Abs_test/Abs_standard) × 5.

The factor 5 corresponds to the concentration of the manufacturer-supplied inorganic phosphorus standard (5 mg/dL), and the formula applies Beer–Lambert proportionality under the linear range of the assay. Measured phosphorus concentrations were converted to ATPase activity units (µmol Pi released per minute per milligram of total protein) using the sample volume, reaction duration, molecular weight of phosphorus (30.97 g/mol), and Bradford-determined protein concentration.

#### 4.7.3. Na^+^/K^+^-ATPase

Cell lysate was added to a reaction mixture containing 90 mM Tris-HCl (pH 7.5), 5 mM MgSO_4_, 20 mM KCl, 100 mM NaCl, 1 mM EDTA, and 5 mM ATP. Samples were incubated at 37 °C for 15 min in a shaking water bath. Reactions were terminated by the addition of 10% trichloroacetic acid (TCA, 200 µL) to precipitate protein and halt enzymatic hydrolysis; samples were then centrifuged at 3000× *g* for 5 min, and the supernatant was used for Pi quantification as described above.

#### 4.7.4. Ca^2+^-ATPase

Reaction mixtures contained 50 mM Tris-HCl (pH 7.5), 5 mM CaCl_2_, 5 mM ATP, and cell lysate. Samples were incubated at 37 °C for 15 min. Reactions were terminated with 10% TCA (200 µL) as described above, and Pi was quantified from the clarified supernatant as described in Section 4.6.2. The Ca^2+^-dependent activity reflects ATP hydrolysis stimulated by the presence of free Ca^2+^ ions, incorporating contributions from plasma membrane Ca^2+^-ATPase (PMCA) and potentially other Ca^2+^-sensitive ATPases.

#### 4.7.5. Mg^2+^-ATPase

Reaction mixtures contained 50 mM Tris-HCl (pH 7.5), 5 mM MgCl_2_, 5 mM ATP, and cell lysate. Incubation (37 °C, 15 min) and termination (10% TCA, 200 µL) were performed identically to the procedures described for Na^+^/K^+^-ATPase and Ca^2+^-ATPase. Pi was quantified from the clarified supernatant as discussed above. Given the universal role of Mg^2+^ as a cofactor in ATP hydrolysis, the Mg^2+^-ATPase activity measurement reflects a composite of multiple ATP-hydrolysing enzymes present in the lysate and should be interpreted as a general indicator of cellular ATP-hydrolysing capacity, as discussed above.

### 4.8. Mitochondrial Membrane Potential Assay

Mitochondrial membrane potential (Δψm) was assessed using the Muse™ MitoPotential Kit (Millipore, Hayward, CA, USA). Following TNF-α pre-exposure (10 ng/mL, 1 h) and carvacrol treatment (6.25 µg/mL, 24 h), cells were harvested by trypsinisation, washed with phosphate-buffered saline, and resuspended at 1 × 10^5^ cells per sample in 1× assay buffer. Cells were stained with 95 µL of MitoPotential working solution (MitoPotential (Hayward, CA, USA) dye at 1:1000 dilution) for 20 min at 37 °C in the dark, followed by addition of 5 µL of Muse™ 7-AAD viability reagent for 5 min at room temperature. Samples were immediately analysed on the Muse™ Cell Analyser using the MitoPotential channel. Results are expressed as percentages of polarised live (high MitoPotential, 7-AAD negative), depolarised live (low MitoPotential, 7-AAD negative), and depolarised dead (low MitoPotential, 7-AAD positive) cells according to manufacturer-defined gating criteria. Representative two-parameter dot plots (MitoPotential dye intensity versus 7-AAD) for each experimental condition are presented alongside quantitative summary data to permit the assessment of gating quality.

### 4.9. Gene Expression Analysis by Quantitative RT-PCR

Total RNA was extracted using TRIzol reagent (Invitrogen, Carlsbad, CA, USA) per manufacturer’s protocol. RNA purity (260/280 ≥ 1.8) was confirmed by NanoDrop spectrophotometry. cDNA was synthesised from 0.5 µg total RNA using the iScript™ cDNA Synthesis Kit (Bio-Rad, Hercules, CA, USA, Cat. #1708891) per manufacturer’s protocol (25 °C 5 min; 46 °C 20 min; 95 °C 1 min). Quantitative PCR was performed in triplicate using SYBR Green Master Mix (G Biosciences, St. Louis, MO, USA, Cat. #786-5062) on a LightCycler 96 system (Roche Diagnostic GbbH, Mannheim, Germany), with the following cycling protocol: 95 °C 2 min; 40 cycles of 95 °C 10 s, 58 °C 60 s, and 72 °C 60 s. Melting curve analysis confirmed amplicon specificity. Gene expression was calculated using the 2^−ΔΔCt^ method relative to GAPDH; GAPDH stability across treatment groups was confirmed (inter-group ΔCt variance < 0.5 cycles). Results are expressed as fold change relative to the TNF-α-treated group. Primer sequences are provided in Table 1.

### 4.10. Statistical Analysis

All experiments were performed as independent biological replicates (n = 3), each conducted on a different day with different cell passages. Technical replicates within each experiment were averaged to generate the single value for each biological replicate. Data are presented as mean ± SEM. Statistical analyses were performed using GraphPad Prism (v6.0; GraphPad Software Inc., San Diego, CA, USA). One-way analysis of variance (ANOVA) was applied to each endpoint with treatment group as the independent variable. Where ANOVA demonstrated significant overall effects (*p* < 0.05), Tukey’s honestly significant difference (HSD) post hoc test was applied to all pairwise comparisons. The Tukey’s correction maintains the family-wise error rate at α = 0.05 across all pairwise tests. Statistical significance thresholds are denoted as: *** *p* < 0.001; ** *p* < 0.01; * *p* < 0.05.

## 5. Conclusions

This study provides a rigorous quantitative mechanistic characterisation of carvacrol’s bioenergetic effects in TNF-α-challenged L6 rat myoblasts. The uniform proportional recovery pattern across three ionically distinct ATPase activities, the structural parallel with LDH recovery, and the mechanistic coherence between Ca^2+^-ATPase restoration and mitochondrial depolarisation attenuation collectively support a membrane-stabilising rather than enzyme-specific mechanism of action. An integrative bioenergetic model is proposed that mechanistically links membrane-ATPase function through Ca^2+^ homeostasis to mitochondrial polarisation, with transcriptional SIRT1/AMPK upregulation as a concurrent adaptive response. The consistency of partial modulation across all endpoints is mechanistically coherent and supports the hypothesis of multi-target engagement by carvacrol under inflammatory conditions. Protein-level validation of SIRT1 and AMPKα, including phospho-AMPK (Thr172), phospho-ACC (Ser79), and SIRT1 deacetylase activity, combined with the inclusion of a carvacrol-only control arm, direct ROS quantification, and differentiated myotube models constitute the essential experimental progressions required to establish causal mechanistic relationships.

## Figures and Tables

**Table 1:** Primer sequences used for quantitative RT-PCR analysis of AMPK, SIRT1, and GAPDH. Tm, primer melting temperature.

Gene	Primer	Sequence (5′–3′)	Temperature (°C)
**AMPK**	Forward	GCTGAGGAACTGGCGGGCG	65.3
	Reverse	GGGAATTAGGTCATAGCAGC	57.3
**SIRT1**	Forward	CGGCTACCGAGGTCCATATAC	61.8
	Reverse	CAGCTCAGGTGGAGGAATTGT	59.8
**GAPDH (reference gene)**	Forward	AATGCATCCTGCACCACCAACTGC	64.4
	Reverse	GGAGGCCATGTAGGCCATGAGGTC	67.8

## Data Availability

The original contributions presented in this study are included in the article. Further inquiries can be directed to the corresponding author.
